# Prevalence of Vitamin D Deficiency and Its Association With Anemia Severity in Anemic Patients: A Cross-Sectional Study

**DOI:** 10.7759/cureus.103157

**Published:** 2026-02-07

**Authors:** Aby Aurthur Michael, Aswathy P T, Deepthi Krishnan

**Affiliations:** 1 Department of General Medicine, Government Medical College Palakkad, Palakkad, IND

**Keywords:** 25-hydroxyvitamin d, anemia, hemoglobin, iron deficiency anemia, vitamin d deficiency

## Abstract

Background

Vitamin D deficiency remains highly prevalent in India, even though the population is exposed to abundant sunlight throughout most of the year. However, data evaluating vitamin D status specifically among anemic adult populations in routine clinical settings remain limited. This study was undertaken to determine the prevalence of vitamin D deficiency in patients with anemia and to examine its relationship with hemoglobin concentration, the severity of anemia, and the underlying anemia subtype.

Methods

This cross-sectional, hospital-based observational study was conducted over six months in the department of general medicine at a tertiary care center in South India. A total of 246 anemic patients (hemoglobin <11 g/dL) aged >13 years were enrolled after obtaining informed consent. Demographic data, body mass index (BMI), sun exposure duration, skin type, and dietary vitamin D intake were recorded. Laboratory evaluation comprised measurement of hemoglobin concentration, serum ferritin, total iron-binding capacity (TIBC), C-reactive protein (CRP), erythrocyte sedimentation rate (ESR), and serum 25-hydroxyvitamin D levels. Vitamin D status was defined based on serum 25-hydroxyvitamin D concentrations and categorized as deficient (<20 ng/mL), insufficient (20-29 ng/mL), or sufficient (≥30 ng/mL). Anemia was subsequently categorized according to both severity and etiological type.

Results

The mean age of the patients was 45.31 ± 18.27 years, with females constituting 156 participants (63.4%). The mean hemoglobin concentration was 8.68 ± 1.81 g/dL. Based on severity, moderate anemia was most common, observed in 121 patients (49.2%), followed by mild anemia in 87 (35.4%) and severe anemia in 38 (15.4%). Iron deficiency anemia was the predominant anemia type, identified in 165 patients (67.1%), while anemia of chronic disease and megaloblastic anemia were present in 71 (28.9%) and 10 patients (4.1%), respectively. The mean serum 25-hydroxyvitamin D level was 23.71 ± 12.00 ng/mL. Vitamin D deficiency was noted in 100 patients (40.7%) and insufficiency in 76 patients (30.9%), resulting in an overall prevalence of suboptimal vitamin D status in 176 patients (71.6%). Mean hemoglobin levels differed significantly across vitamin D status categories (ANOVA, p = 0.017) and dietary vitamin D intake categories (ANOVA, p = 0.008). Analysis revealed a statistically significant association between vitamin D status and the severity of anemia (χ² = 16.01, p = 0.003). In contrast, vitamin D status showed no significant relationship with the etiological classification of anemia (p = 0.531). Similarly, dietary vitamin D intake was not significantly associated with either anemia severity or anemia type. Pearson’s correlation analysis indicated the absence of a meaningful linear relationship between serum vitamin D concentrations and hemoglobin levels (r = 0.050, p = 0.434).

Conclusion

Vitamin D deficiency is highly prevalent among anemic patients. Although serum vitamin D levels did not show a significant linear correlation with hemoglobin concentration, categorical vitamin D status was associated with anemia severity in a non-linear manner, highlighting the potential clinical relevance of vitamin D status in anemic populations.

## Introduction

Vitamin D is a fat-soluble secosteroid that is primarily obtained through endogenous production rather than dietary intake. Approximately 90% of the body’s vitamin D needs are met through endogenous production in the skin after exposure to ultraviolet B (UV-B) radiation, within a wavelength range of 290-320 nm. Dietary contribution to vitamin D levels is relatively limited and is mainly derived from fatty fish, fortified food products, and pharmacological supplements, whereas plant-based foods and cereals provide minimal amounts of this vitamin [1].

Vitamin D plays a vital role in several physiological processes essential for normal body function. Traditionally, it is recognized for its role in facilitating intestinal calcium absorption and maintaining overall mineral balance. In addition to calcium metabolism, vitamin D contributes to the regulation of other important minerals, including iron, zinc, magnesium, and phosphorus. The biologically active form of vitamin D also participates in the modulation of parathyroid hormone (PTH) secretion. Persistent elevation of PTH levels can accelerate bone resorption, leading to reduced bone strength and an increased susceptibility to osteoporosis [2].

Vitamin D deficiency is now recognized as a major public health issue affecting populations across the globe, with more than one billion individuals estimated to have suboptimal levels. Notably, a high burden of vitamin D deficiency has been documented in India despite its tropical climate and ample sunlight exposure. Evidence from several regional and population-based studies indicates that the prevalence of vitamin D deficiency in India varies widely, with reported rates ranging from approximately 34% to as high as 94% across different demographic groups [1].

Low serum concentrations of 25-hydroxyvitamin D have been linked to a wide spectrum of adverse health outcomes. While its deficiency is well known to affect skeletal integrity, contributing to conditions such as osteoporosis, fractures, and altered bone metabolism, it has also been implicated in several extra-skeletal disorders. These include metabolic and autoimmune conditions such as type 1 diabetes mellitus and multiple sclerosis, cardiovascular and infectious diseases, as well as neuropsychiatric disorders like depression and schizophrenia. Additionally, vitamin D deficiency has been associated with obesity and chronic respiratory illnesses, including chronic obstructive pulmonary disease [3].

Emerging evidence indicates that vitamin D deficiency may be linked to a higher likelihood of anemia, a major public health concern that affects a substantial proportion of the population. In India, anemia remains highly prevalent, particularly among children, with reports suggesting that nearly half of this group may be affected [4]. Experimental and clinical studies have demonstrated that vitamin D deficiency may be associated with increased expression of hepcidin at the mRNA level. Elevated hepcidin activity leads to reduced availability of ferroportin, the key iron-export protein involved in intestinal iron absorption and systemic iron regulation. Consequently, sufficient serum vitamin D levels may help suppress excessive hepcidin expression, thereby supporting iron homeostasis and potentially reducing the risk of iron deficiency [5]. Vitamin D receptors have been identified within the bone marrow, where their expression is markedly higher than in circulating plasma, suggesting a potential role for vitamin D in bone marrow-mediated hematopoietic processes [6]. In addition to its regulatory effects on mineral metabolism, vitamin D appears to play a contributory role in erythropoiesis, and emerging evidence suggests that vitamin D supplementation may support the management of anemia in selected patient populations [7].

Despite these findings, the relationship between vitamin D deficiency and anemia remains incompletely understood. Many studies are observational in nature, limiting the ability to establish causality. Additionally, confounding factors such as dietary habits, sun exposure, and comorbidities may influence the observed associations. There is a critical need for well-designed studies to assess the prevalence of vitamin D deficiency among anemic patients and to explore the underlying mechanisms linking these two conditions.

This study aims to address this gap by assessing the prevalence of vitamin D deficiency in anemic patients and examining the correlation between serum 25(OH)D levels and hemoglobin concentrations. By leveraging existing clinical data and employing rigorous statistical analyses, this study seeks to provide insights into the potential role of vitamin D in the development and management of anemia. The findings of this study may inform clinical practice by highlighting the importance of screening for vitamin D deficiency in anemic patients and exploring the potential benefits of vitamin D supplementation as part of a comprehensive treatment strategy.

In conclusion, understanding the relationship between vitamin D deficiency and anemia has significant clinical and public health implications. This study contributes to the growing body of evidence on this topic and underscores the need for further research to elucidate the mechanisms underlying this association and to evaluate the potential therapeutic benefits of vitamin D in anemic patients.

## Materials and methods

Study design and setting

This study employed a cross-sectional, hospital-based observational design and was carried out at a single tertiary care center over a six-month period, from July to December 2025, in the Department of General Medicine at Government Medical College, Palakkad. The study has been approved by the Institutional Ethics Committee (IEC) of Government Medical College Palakkad, India (IEC/GMCPKD/14/2025/164), and written informed consent was secured from all participants before their inclusion in the study.

Study population

Consecutive patients presenting with anemia during the study period were screened for eligibility. Anemia was defined as a hemoglobin concentration of less than 11 g/dL. Eligible patients fulfilling the inclusion and exclusion criteria were enrolled until the required sample size was achieved. A total of 246 anemic patients were included in the final analysis.

Sample size calculation

The required sample size was determined using a standard prevalence-based formula [8]:

$n = Z^2 \times p \times (1-p) / d^2$

In this equation, n denotes the minimum sample size, Z represents the standard normal value corresponding to a 95% confidence interval (1.96), p indicates the anticipated prevalence of vitamin D deficiency among anemic patients (assumed to be 20%), and d refers to the permissible margin of error, set at 5%. Based on these parameters, the final calculated sample size was 246 participants.

Inclusion and exclusion criteria

Patients aged more than 13 years of either sex with hemoglobin levels below 11 g/dL and willing to participate in the study were included. Patients with chronic kidney disease, chronic liver disease, malignancy, or known hematological disorders such as aplastic anemia, sickle cell anemia, thalassemia, myelodysplastic syndromes, or hemolytic anemia were excluded. Patients receiving vitamin D, calcium, folic acid, or vitamin B12 supplementation, as well as those on anticonvulsants, systemic steroids, or orlistat, were also excluded to minimize confounding effects.

Definitions

The severity of anemia was graded according to hemoglobin concentration and classified as mild (10-10.9 g/dL), moderate (7-9.9 g/dL), or severe (<7 g/dL) [9]. Etiological classification of anemia was performed using relevant hematological and biochemical findings, categorizing cases as iron deficiency anemia, anemia of chronic disease, or megaloblastic anemia. Serum 25-hydroxyvitamin D concentrations were used to define vitamin D status, with levels <20 ng/mL considered deficient, 20-29 ng/mL considered insufficient, and values ≥30 ng/mL regarded as sufficient [10].

Data collection

Baseline demographic variables, including age and sex, were documented for all enrolled participants. Anthropometric measurements were obtained to compute body mass index (BMI), which was calculated as body weight in kilograms divided by the square of height in meters. Based on standard World Health Organization criteria, BMI was classified into four categories based on standard cut-off values: underweight (<18.5 kg/m²), normal weight (18.5-24.9 kg/m²), overweight (25.0-29.9 kg/m²), and obese (≥30.0 kg/m²) [11]. Lifestyle factors such as average daily sun exposure duration were documented in minutes, and skin pigmentation was classified using Fitzpatrick skin types [12]. Dietary vitamin D intake was assessed using dietary history and categorized as low, moderate, or high.

Laboratory investigations

Venous blood samples were collected from all participants under aseptic precautions. Hemoglobin concentration was measured using the HemoCue AB system (Ängelholm, Sweden). Serum ferritin, total iron-binding capacity (TIBC), C-reactive protein (CRP), and erythrocyte sedimentation rate (ESR) were measured using standard laboratory techniques. Serum 25-hydroxyvitamin D levels were measured using a quantitative electro-chemiluminescence immunoassay. All laboratory investigations were performed in the central laboratory following standard operating procedures [13].

Statistical analysis

Data analysis was carried out using IBM SPSS Statistics for Windows, version 27.0 (IBM Corp., Armonk, NY). Continuous variables were summarized as means with standard deviations, whereas categorical variables were described using frequencies and percentages. Normality of continuous variables was assessed prior to analysis. For variables with wide dispersion, results are presented as median (interquartile range) in addition to mean ± standard deviation. Comparisons of mean hemoglobin levels across vitamin D status and dietary vitamin D intake categories were performed using one-way analysis of variance (ANOVA). Associations between categorical variables, including vitamin D status, with anemia severity and anemia type were assessed using the chi-square test. Pearson correlation analysis was used for correlation analysis. Statistical significance was defined as a p-value less than 0.05.

## Results

A total of 246 anemic patients were included in the study, with a mean age of 45.31 ± 18.27 years. The largest age group comprised patients aged 41-60 years (80, 32.5%), followed by those aged 21-40 years (74, 30.1%), 61-80 years (64, 26.0%), and ≤20 years (28, 11.4%). Females constituted the majority of the study population (156, 63.4%), while males accounted for 90 participants (36.6%) (Table 1).

**Table 1:** Baseline demographic, anthropometric, lifestyle, and laboratory characteristics of the study population (n = 246).

Variable	Domain	Mean ± SD or N (%)
Age group	Mean age	45.31 ± 18.27 years
	<=20 years	28 (11.4%)
	21-40 years	74 (30.1%)
	41-60 years	80 (32.5%)
	61-80 years	64 (26%)
Gender	Male	90 (36.6%)
	Female	156 (63.4%)
BMI [11]	Mean BMI	23.75 ± 4.090
	Underweight	23 (9.3%)
	Normal weight	125 (50.8%)
	Overweight	82 (33.3%)
	Obese	16 (6.5%)
Sun exposure	Mean duration	71.44 ± 43.31 min
	Median duration	66.00 (36.00-108.00)
	0-30 min	52 (21.1%)
	31-60 min	59 (24%)
	61-90 min	59 (24%)
	91-120 min	46 (18.7%)
	121-150 min	22 (8.9%)
	151-180 min	8 (3.3%)
Skin type [12]	Type III	42 (17.1%)
	Type IV	70 (28.5%)
	Type V	98 (39.8%)
	Type VI	36 (14.6%)
Ferritin	Mean	57.79 ± 71.06 ng/ml
	Median	16.15 (9.50–117.10)
Total iron-binding capacity (TIBC)	Mean	347.02 ± 108.66 µg/dl
	Median	389.7 (227.0–435.2)
C-reactive protein (CRP)	Mean	17.03 ± 7.34 mg/l
	Median	17.50 (11.20–23.20)
Erythrocyte sedimentation rate (ESR)	Mean	41.03 ± 15.85 mm/hr
	Median	40.00 (28.00–55.00)

The mean body mass index (BMI) was 23.75 ± 4.09 kg/m². Most participants had a normal BMI (125, 50.8%), while 82 (33.3%) were overweight, 23 (9.3%) were underweight, and 16 (6.5%) were obese. The mean daily sun exposure duration was 71.44 ± 43.31 minutes, with 52 participants (21.1%) reporting 0-30 minutes of exposure and 59 participants each reporting 31-60 minutes (24.0%) and 61-90 minutes (24.0%) of daily sunlight exposure (Table 1).

Regarding skin pigmentation, Fitzpatrick skin type V was most common (98, 39.8%), followed by type IV (70, 28.5%), type III (42, 17.1%), and type VI (36, 14.6%). Baseline laboratory evaluation showed a mean serum ferritin level of 57.79 ± 71.06 ng/mL and a mean total iron-binding capacity (TIBC) of 347.02 ± 108.66 µg/dL. Mean CRP and ESR levels were 17.03 ± 7.34 mg/L and 41.03 ± 15.85 mm/hr, respectively (Table 1).

The mean hemoglobin concentration of the study population was 8.68 ± 1.81 g/dL. Based on severity grading, nearly half of the patients had moderate anemia (121, 49.2%), followed by mild anemia in 87 patients (35.4%) and severe anemia in 38 patients (15.4%). With respect to etiology, iron deficiency anemia was the most common type, observed in 165 patients (67.1%). Anemia of chronic disease was identified in 71 patients (28.9%), while megaloblastic anemia was present in 10 patients (4.1%) (Table 2).

**Table 2:** Anemia profile of the study population (n = 246). IDA: iron deficiency anemia; ACD: anemia of chronic disease.

Variable	Domain	Mean ± SD or N (%)
Anemia status	Mean hemoglobin	8.68 ± 1.81 g/dl
Anemia severity [9]	Mild	87 (35.4%)
	Moderate	121 (49.2%)
	Severe	38 (15.4%)
Anemia type [9]	IDA	165 (67.1%)
	ACD	71 (28.9%)
	Megaloblastic	10 (4.1%)

The mean serum 25-hydroxyvitamin D level of the study population was 23.71 ± 12.00 ng/mL. Vitamin D deficiency was observed in 100 patients (40.7%), while 76 patients (30.9%) had vitamin D insufficiency and 70 patients (28.5%) had sufficient vitamin D levels. Overall, 176 participants (71.6%) had suboptimal vitamin D status, defined as deficient or insufficient levels. Assessment of dietary vitamin D intake revealed that a majority of patients had low dietary intake (146, 59.3%), whereas 86 patients (35.0%) reported moderate intake, and only 14 patients (5.7%) reported high dietary vitamin D intake (Table 3).

**Table 3:** Vitamin D status and dietary vitamin D intake among the study population (n = 246).

Variable	Domain	Mean ± SD or N (%)
Vitamin D	Mean vitamin D	23.71 ± 12.00 ng/ml
Vitamin D status [10]	Deficient	100 (40.7%)
	Insufficient	76 (30.9%)
	Sufficient	70 (28.5%)
Dietary vitamin D	Low	146 (59.3%)
	Moderate	86 (35%)
	High	14 (5.7%)

The mean hemoglobin levels differed significantly across categories of vitamin D status (one-way ANOVA, p = 0.017). Patients with vitamin D deficiency had a mean hemoglobin level of 8.69 ± 1.96 g/dL, while those with vitamin D insufficiency and sufficiency had mean hemoglobin levels of 8.38 ± 1.60 g/dL and 8.68 ± 1.82 g/dL, respectively. A statistically significant difference in mean hemoglobin levels was also observed across dietary vitamin D intake categories (one-way ANOVA, p = 0.008). Patients with low dietary vitamin D intake had a mean hemoglobin level of 8.64 ± 1.83 g/dL, compared with 8.84 ± 1.79 g/dL among those with moderate intake and 8.15 ± 1.72 g/dL among those with high intake (Table 4).

**Table 4:** Comparison of mean hemoglobin levels across vitamin D status and dietary vitamin D intake (n = 246).

Variable	Domain	Hemoglobin (Mean ± SD)	P-value (ANOVA)
Vitamin D status [10]	Deficient	8.69 ± 1.96	p=0.017
	Insufficient	8.38 ± 1.60	
	Sufficient	8.68 ± 1.815	
Dietary vitamin D	Low	8.64 ± 1.83	p=0.008
	Moderate	8.84 ± 1.79	
	High	8.15 ± 1.72	

A statistically significant association was observed between vitamin D status and anemia severity (χ² = 16.01, p = 0.003). Among patients with vitamin D deficiency, mild, moderate, and severe anemia were observed in 38 patients (38.0%), 46 patients (46.0%), and 16 patients (16.0%), respectively. In contrast, patients with vitamin D insufficiency predominantly had moderate anemia (50, 65.8%), while those with sufficient vitamin D levels most commonly had mild anemia (34, 48.6%). No statistically significant association was found between vitamin D status and anemia type (χ² = 3.16, p = 0.531). Iron deficiency anemia was the most common anemia type across all vitamin D categories, observed in 66 patients (66.0%) with vitamin D deficiency, 50 patients (65.8%) with insufficiency, and 49 patients (70.0%) with sufficient vitamin D levels. Anemia of chronic disease and megaloblastic anemia were less frequent and showed comparable distributions across the vitamin D status groups (Table 5).

**Table 5:** Association between vitamin D status and anemia severity and type (n = 246). IDA: iron deficiency anemia; ACD: anemia of chronic disease.

Vitamin D status		Deficient	Insufficient	Sufficient	Statistics
Anemia severity [9]	Mild anemia	38 (38%)	15 (19.7%)	34 (48.6%)	χ^2^=16.01; p=0.003
	Moderate anemia	46 (46%)	50 (65.8%)	25 (35.7%)	
	Severe anemia	16 (16%)	11 (14.5%)	11 (15.7%)	
Anemia type [9]	IDA	66 (66%)	50 (65.8%)	49 (70%)	χ^2^=3.16; p=0.531
	ACD	28 (28%)	25 (32.9%)	18 (25.7%)	
	Megaloblastic	6 (6%)	1 (1.3%)	3 (4.3%)	

No statistically significant association was observed between dietary vitamin D intake and anemia severity (χ² = 2.19, p = 0.700). Among patients with low dietary vitamin D intake, mild, moderate, and severe anemia were present in 50 patients (34.2%), 73 patients (50.0%), and 23 patients (15.8%), respectively. Similar distributions were noted among those with moderate dietary intake, with mild anemia in 34 patients (39.5%), moderate anemia in 39 patients (45.3%), and severe anemia in 13 patients (15.2%). In patients reporting high dietary vitamin D intake, moderate anemia was most common (9, 64.3%), while mild and severe anemia were observed in three (21.4%) and two patients (14.3%), respectively. Similarly, dietary vitamin D intake was not significantly associated with anemia type (χ² = 4.79, p = 0.310). Iron deficiency anemia was the predominant anemia type across all dietary intake categories, observed in 100 patients (68.5%) with low intake, 55 patients (64.0%) with moderate intake, and 10 patients (71.4%) with high intake. The proportions of anemia of chronic disease and megaloblastic anemia were comparable across the dietary vitamin D intake groups (Table 6).

**Table 6:** Association between dietary vitamin D intake and anemia severity and type (n = 246). IDA: iron deficiency anemia; ACD: anemia of chronic disease.

Dietary vitamin D		Low	Moderate	High	Statistics
Anemia severity [9]	Mild anemia	50 (34.2%)	34 (39.5%)	3 (21.4%)	χ^2^=2.19; p=0.700
	Moderate anemia	73 (50%)	39 (45.3%)	9 (64.3%)	
	Severe anemia	23 (15.8%)	13 (15.2%)	2 (14.3%)	
Anemia type [9]	IDA	100 (68.5%)	55 (64%)	10 (71.4%)	χ^2^=4.79; p=0.310
	ACD	38 (26%)	30 (34.9%)	3 (21.4%)	
	Megaloblastic	8 (5.5%)	1 (1.2%)	1 (7.1%)	

**Figure 1:**
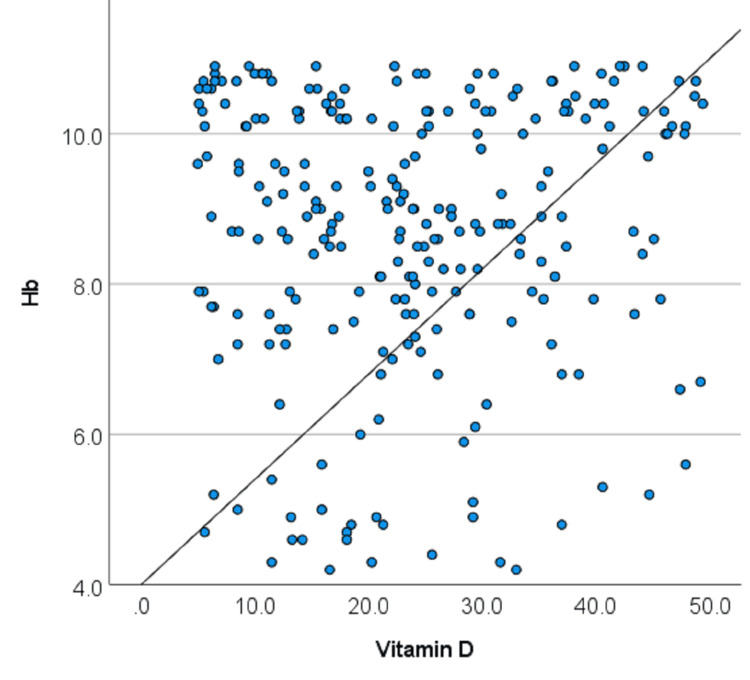
Scatter plot showing the relationship between serum 25-hydroxyvitamin D levels and hemoglobin (Hb) concentration (n = 246).

Pearson correlation analysis demonstrated a weak positive correlation between serum vitamin D levels and hemoglobin concentration (r = 0.050); however, this association was not statistically significant (p = 0.434), indicating no meaningful linear relationship between vitamin D levels and hemoglobin concentration in the study population (Figure 1).

## Discussion

The present cross-sectional study demonstrates a high prevalence of vitamin D deficiency among anemic patients and identifies a significant association between vitamin D status and anemia severity. These findings are consistent with a growing body of observational evidence suggesting that vitamin D deficiency and anemia frequently coexist across different age groups and clinical settings.

Sim et al. reported a significantly higher prevalence of anemia among individuals with vitamin D deficiency compared with those having sufficient vitamin D levels in a large integrated healthcare population [14]. They further demonstrated lower mean hemoglobin levels and an increased likelihood of anemia among vitamin D-deficient subjects. In the present study, although a significant linear correlation between serum vitamin D levels and hemoglobin concentration was not observed, categorical analysis revealed significantly lower mean hemoglobin levels and greater anemia severity among vitamin D-deficient patients, supporting a non-linear association as previously suggested [14].

Age and sex distributions varied across studies but did not alter the observed relationship. While Sim et al. and Perlstein et al. primarily evaluated elderly populations [14,15], our study included a broader adult age range with female predominance. Despite these differences, all studies consistently demonstrated a higher burden of vitamin D deficiency among anemic individuals, indicating that this association is not limited to elderly populations alone.

Lifestyle-related determinants of vitamin D status, including sun exposure and skin pigmentation, were specifically assessed in the present study. Pediatric studies by Yoon et al. and Chowdhury et al. similarly highlighted the role of reduced sun exposure and skin pigmentation as contributors to vitamin D deficiency among children with iron deficiency anemia [4,16]. Although these factors were not uniformly assessed in adult cohorts, the consistency of findings across age groups supports the multifactorial nature of vitamin D deficiency.

Iron indices offer important insight into the relationship between vitamin D deficiency and anemia. Sim et al. and Perlstein et al. observed higher ferritin levels and lower TIBC among vitamin D-deficient individuals, suggesting that iron deficiency alone did not fully account for anemia and raising the possibility of anemia of inflammation [14,15]. In our study, iron deficiency anemia was the most common anemia type across all vitamin D categories, and no significant association was observed between vitamin D status and anemia type. However, elevated inflammatory markers such as CRP and ESR in our cohort are consistent with observations linking vitamin D deficiency, inflammation, and anemia severity [15].

Pediatric studies have shown a high prevalence of vitamin D deficiency among children with iron deficiency anemia, though associations with hemoglobin levels have been variable. Yoon et al. reported no significant difference in hemoglobin levels despite a high prevalence of vitamin D deficiency [16], while Chowdhury et al. observed an association between vitamin D deficiency and moderate anemia but not overall anemia prevalence [4]. These findings parallel our observation that vitamin D status was associated with anemia severity rather than anemia type or hemoglobin concentration alone.

Indian adult data further support the coexistence of vitamin D deficiency and anemia. Kumari et al. reported a significantly higher prevalence of anemia and lower mean hemoglobin levels among vitamin D-deficient adults [17]. Our findings are concordant with this observation, although the absence of a significant linear correlation in our study highlights the complexity of this association.

Studies in specific populations have also demonstrated similar associations. Kartal et al. reported lower hemoglobin levels and a positive correlation between vitamin D and hemoglobin among anemic pregnant women [18], while Moslhy et al. observed worsening iron deficiency and elevated inflammatory markers in vitamin D-deficient children with type 1 diabetes mellitus [19]. Although such populations were excluded from the present study, elevated inflammatory markers in our cohort suggest that inflammatory pathways may contribute to anemia severity in vitamin D-deficient individuals.

The strengths of the present study include a well-characterized anemic population, assessment of multiple demographic, lifestyle, and laboratory parameters, and standardized classification of vitamin D status and anemia severity. However, similar to prior studies, the cross-sectional design precludes causal inference, and residual confounding cannot be excluded.

A uniform hemoglobin cutoff of <11 g/dL was applied to both sexes to ensure consistent severity grading; however, this approach may have excluded adult males with mild anemia (hemoglobin = 11-13 g/dL), thereby limiting generalizability to this subgroup. Although key determinants such as BMI, sun exposure, dietary vitamin D intake, and skin pigmentation were assessed, detailed evaluation of socioeconomic status, ethnicity, and iron-specific dietary intake was not performed, and residual confounding cannot be excluded. Some continuous variables demonstrated wide dispersion, and while parametric tests were employed, with ANOVA being generally robust in large samples, data skewness may have influenced effect estimates. Multivariate regression analysis was not undertaken, as the study was designed to be descriptive and exploratory; consequently, independent associations between vitamin D status and anemia severity after adjustment for confounders could not be established. Additionally, although low dietary vitamin D intake was common, iron intake was not assessed separately, limiting conclusions regarding shared dietary pathways contributing to concurrent iron and vitamin D deficiency. Future prospective studies incorporating detailed dietary assessment and multivariable modeling are warranted.

## Conclusions

This study demonstrates a high prevalence of vitamin D deficiency among anemic patients in a tertiary care setting. While serum vitamin D levels did not show a significant linear correlation with hemoglobin concentration, categorical vitamin D status was associated with anemia severity in a non-linear manner. These findings suggest that categorical vitamin D status may have clinical relevance in the evaluation of anemia, even in the absence of a direct linear relationship with hemoglobin levels. Routine assessment of vitamin D status in anemic patients may help identify individuals at risk of more severe disease. Further prospective and interventional studies are required to clarify causal relationships and to evaluate the potential role of vitamin D supplementation in anemia management.
